# Sphingosine 1-Phosphate Receptor 5 (S1P_5_) Knockout Ameliorates Adenine-Induced Nephropathy

**DOI:** 10.3390/ijms23073952

**Published:** 2022-04-02

**Authors:** Timon Eckes, Sammy Patyna, Alexander Koch, Anke Oftring, Stefan Gauer, Nicholas Obermüller, Stephanie Schwalm, Liliana Schaefer, Jerold Chun, Hermann-Josef Gröne, Josef Pfeilschifter

**Affiliations:** 1 Institute of General Pharmacology and Toxicology, University Hospital Frankfurt, Goethe University Frankfurt am Main, 60590 Frankfurt am Main, Germany; patyna@em.uni-frankfurt.de (S.P.); alexander.koch@med.uni-frankfurt.de (A.K.); oftring@med.uni-frankfurt.de (A.O.); s.schwalm@med.uni-frankfurt.de (S.S.); schaefer@med.uni-frankfurt.de (L.S.); pfeilschifter@em.uni-frankfurt.de (J.P.); 2 Department of Nephrology, University Hospital Frankfurt, Goethe University Frankfurt am Main, 60590 Frankfurt am Main, Germany; stefan.gauer@kgu.de (S.G.); nicholas.obermueller@kgu.de (N.O.); 3 Sanford Burnham Prebys Medical Discovery Institute, La Jolla, CA 92037, USA; jchun@sbpdiscovery.org; 4 Institute of Pharmacology, Philipps-University, 35037 Marburg, Germany; hermann-josef.groene@uni-marburg.de

**Keywords:** sphingolipids, lysophospholipids, S1P, S1P_5_, S1PR5, fibrosis, nephropathy, renal inflammation

## Abstract

S1P and its receptors have been reported to play important roles in the development of renal fibrosis. Although S1P_5_ has barely been investigated so far, there are indications that it can influence inflammatory and fibrotic processes. Here, we report the role of S1P_5_ in renal inflammation and fibrosis. Male S1P_5_ knockout mice and wild-type mice on a C57BL/6J background were fed with an adenine-rich diet for 7 days or 14 days to induce tubulointerstitial fibrosis. The kidneys of untreated mice served as respective controls. Kidney damage, fibrosis, and inflammation in kidney tissues were analyzed by real-time PCR, Western blot, and histological staining. Renal function was assessed by plasma creatinine ELISA. The S1P_5_ knockout mice had better renal function and showed less kidney damage, less proinflammatory cytokine release, and less fibrosis after 7 days and 14 days of an adenine-rich diet compared to wild-type mice. S1P_5_ knockout ameliorates tubular damage and tubulointerstitial fibrosis in a model of adenine-induced nephropathy in mice. Thus, targeting S1P_5_ might be a promising goal for the pharmacological treatment of kidney diseases.

## 1. Introduction

Chronic kidney disease (CKD) is a prominent health problem with a prevalence of almost 15% of all adults in the U.S., and with a varying prevalence in Europe of 3.3% in Norway and 17.3% in the northeast of Germany based on studies published in 2019 and 2016, respectively [1,2]. Pathophysiological hallmarks are glomerular and tubular injury, enhanced synthesis and secretion of cytokines and growth factors, and increased formation of extracellular matrix, leading to glomerulosclerosis and tubulointerstitial fibrosis. The subsequent loss of renal function is reflected by the decline of the glomerular filtration rate and albuminuria, and finally results in the need for dialysis or kidney transplantation. 

Interestingly, there is growing evidence that the pleiotropic lipid mediator sphingosine 1-phosphate (S1P) is able to affect the progression of CKD [3,4]. S1P belongs to the sphingolipid family of signaling mediators and can be generated by the phosphorylation of sphingosine by either sphingosine kinase (SK)-1 or SK-2 [5]. Once generated intracellularly, it can act on targets within the cell or be secreted via specific transporters to activate the G protein-coupled S1P receptors (S1P_1–5_) on the plasma membrane [6,7,8]; S1P receptors are coupled to different G proteins, such as G_i_, G_q_ or G_12/13_, to mediate various signals and show a cell type and cell status-dependent expression pattern [9,10,11,12,13]. Modulation of S1P receptors can improve renal disease outcomes, as shown in previous studies [3]. Chronic pharmacological S1P_1_ activation by treatment with FTY720 or SEW2871 was found to ameliorate albuminuria and renal damage in a model of diabetic nephropathy in rats [14]. S1P_1_ activation was also protective against renal ischemia-reperfusion injury in mice [15]. 

S1P_5_ (formerly denoted as edg-8) was the last S1P receptor to be discovered and is coupled to G_i_ and G_12_ [16]. Its expression was seen predominantly in oligodendrocytes and fibrous astrocytes in rat brains [17]. Further studies showed that S1P_5_ expression can be found on various cell types, e.g., subsets of monocytes, natural killer (NK) cells, dendritic cells, and cancer cells [18,19,20,21]. However, S1P_5_ expression is not ubiquitous, as is described for S1P_1–3_ [18,19,20,22]. Still, there is little known about the relevance and function of S1P_5_ in physiology and disease. Previously, we found S1P_5_ expression in human renal mesangial cells, which is further inducible by stimulation with transforming growth factor-beta 2 (TGF-β_2_) [23]. Most interestingly, downregulation of S1P_5_ by siRNA impaired the TGF-β_2_-induced expression of fibrotic connective tissue growth factor (CTGF) in human mesangial cells, indicating a potential role of S1P_5_ in the progression of fibrosis [23]. A second hint was found in a model of bleomycin-induced systemic sclerosis, where the skin tissue of S1P_5_ knockout mice (S1P_5_^-/-^) showed significant lower mRNA levels of profibrotic collagen 1 alpha 1 (Col1α1) compared to wild-type mice [24]. 

Taken together, these data suggest that S1P_5_ might play a role not only in the in vitro regulation of fibrotic mediators in renal cells [23], but also for the progression of kidney diseases in vivo. Thus, we investigated S1P_5_^-/-^ and corresponding, wild-type mice in a model of adenine-induced nephropathy to evaluate the function of S1P_5_ for the progression of tubular injury and tubulointerstitial fibrosis. 

## 2. Results

### 2.1. S1P_5_ Was Upregulated in Human Fibrotic Renal Tissue

**Figure 1:**
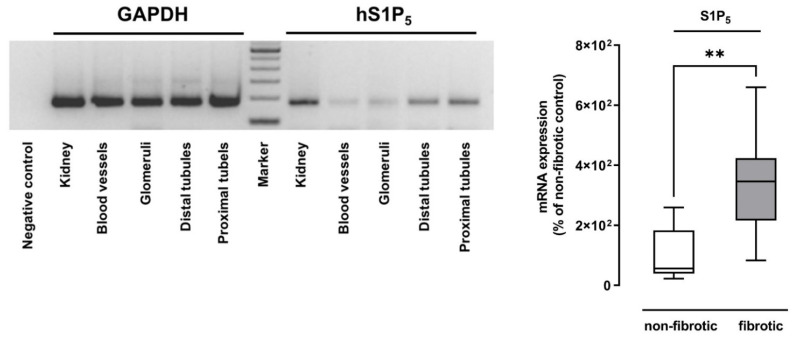
Expression of S1P_5_ in human kidney samples. PCR of S1P_5_ mRNA expression in human kidney and kidney tissue sections (left side) and quantification from RT-PCR of S1P_5_ mRNA in non-fibrotic and fibrotic kidney tissue of patients suffering from hydronephrosis (right side). GAPDH was used as a control in both experiments. Data are shown as box plots with a line at the median and whiskers from minimum to maximum value; *n* = 5 (non-fibrotic), *n* = 10 (fibrotic), ** *p* < 0.01; Mann–Whitney U test.

In a previous study, we demonstrated that the downregulation of S1P_5_ impairs the TGF-β_2_-mediated expression of profibrotic CTGF in human mesangial cells [23]. Here, we report that S1P_5_ is also upregulated in the fibrotic tissue of patients suffering from hydronephrosis (Figure 1). Semi-quantitative PCR analysis of human kidney samples revealed that S1P_5_ is mainly expressed in tubular cells (Figure 1). Hence, we further investigated the involvement of S1P_5_ in renal fibrosis.

### 2.2. S1P_5_ Knockout Mice Had Less Tissue Damage and Showed Better Kidney Function after Disease Induction

To elucidate the role of S1P_5_ in renal fibrosis, we employed an in vivo model of tubulointerstitial fibrosis induced by sterile inflammation. Wild-type and S1P_5_^-/-^ mice, both on a C57BL/6J background, were fed for either 7 or 14 days with an adenine-rich diet (0.25% adenine), which led to the deposition of insoluble, crystal-forming 2,8-dihydroxyadenine in the kidney, which was able to damage the surrounding tissue as described in the literature [25]. 

**Figure 2:**
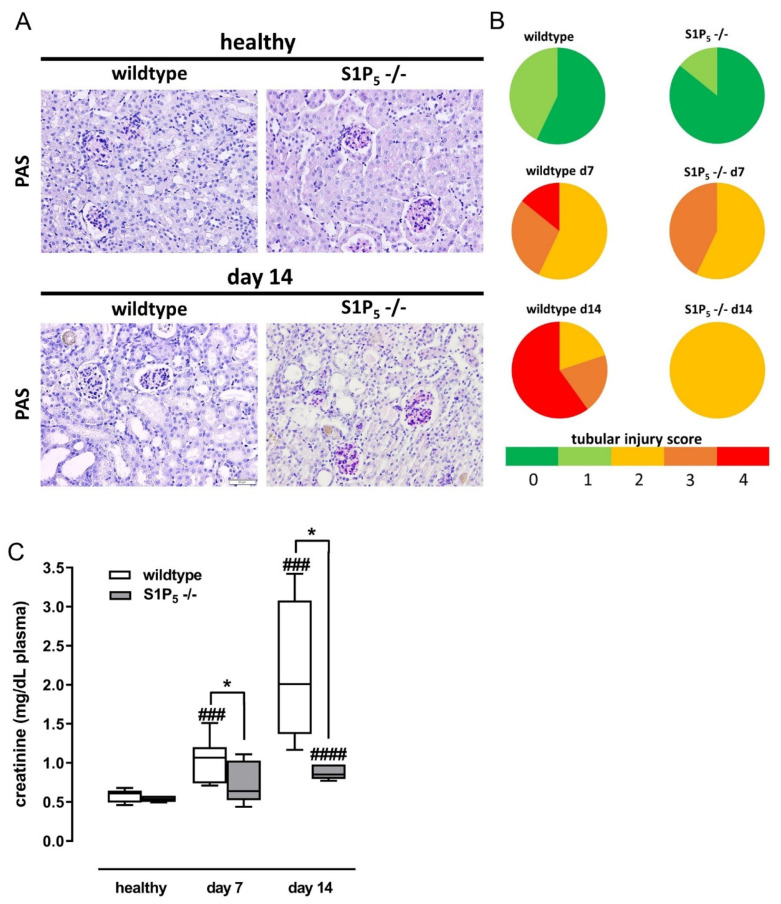
Assessment of renal tissue damage and plasma creatinine levels of healthy mice and mice fed with an adenine-rich diet for the indicated time periods. (**A**) Paraffin-embedded kidney cortex sections were subjected to PAS staining. The images reveal glomeruli (dark pink), tubular dilatations (big gaps), and crystal depositions (amber color). (**B**) Tissue damage of wild-type and S1P_5_ knockout mice (S1P_5_^-/-^) without (healthy) and with adenine-rich diet for 7 days and 14 days were evaluated by three blinded observers according to the following tubular injury score: 0/dark green: 0%; 1/light green: ≤25%; 2/yellow: >25% to 50%; 3/orange: ≥50% to <75%; 4/red: ≥75% of the area displayed tubular damage. (**C**) Creatinine levels were measured in plasma samples (EDTA) by ELISA as described in the Materials and Methods Section. Data are shown as box plots with a line at the median and whiskers from minimum to maximum value; * *p* ≤ 0.05 as indicated; ### *p* ≤ 0.001, #### *p* ≤ 0.0001 as compared to the respective healthy controls.

Periodic acid-Schiff (PAS) staining of the kidney tissue revealed that the mice of both genotypes suffered from severe tissue damage induced by the adenine treatment (Figure 2A). Tubules were dilatated and necrotic, and amber-colored crystals appeared. The tubular injury of the PAS-stained slides was semi-quantified by three blinded observers employing a four-point scale (Figure 2B). Ten to fifteen randomly chosen cortex regions were rated by assessing tubular dilatation and necrosis. The crystals were not visible in every kidney slide and were only visible after 14 days of adenine treatment even though the tissue was found to be damaged after only 7 days. This is probably due to washout; crystals only remain if they have had enough time to achieve a certain size. However, counting the number of crystals that were still present in kidney slides, we found no difference between wild-type and knockout mice (data not shown). 

The focus of this study was on the interstitial compartment. Nevertheless, we also checked for glomerular lesions after 14 days of the adenine-rich diet. We observed a minor level of sclerotic glomerular lesions in wild-type and knockout mice without significant difference between the groups (*p* = 0.117).

To assess the functionality of the kidneys, we measured plasma creatinine levels and found a significant increase in wild-type mice after 7 and 14 days (Figure 2C). For S1P_5_ knockout mice, however, a significant increase was only present after 14 days. Looking at the difference between the two genotypes, we saw that creatinine levels were similar in healthy mice but significantly lower in diseased S1P_5_^-/-^ mice after 7 days and 14 days of the adenine-rich diet. 

### 2.3. S1P_5_ Knockout Mice Expressed Lower Levels of Kidney Injury Markers after Disease Induction

**Figure 3:**
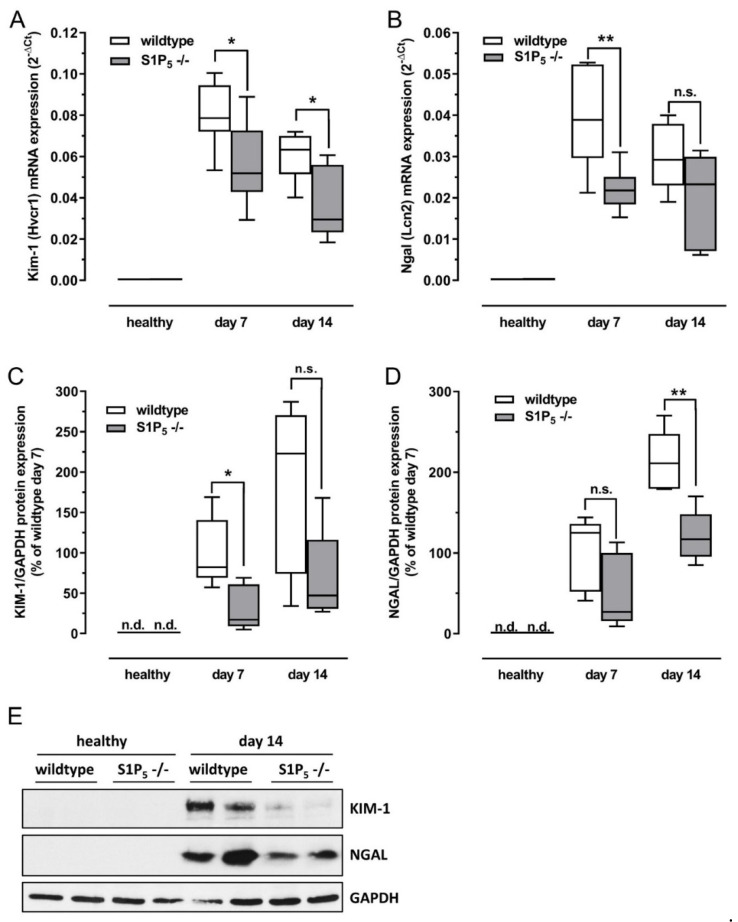
Expression of tissue damage markers in kidney homogenates of healthy mice and mice fed with an adenine-rich diet for the indicated time periods. The expression was compared between wild-type mice (C57BL/6J) and S1P_5_ knockout mice (S1P_5_^-/-^). (**A**,**B**) Expression of Kim-1 and Ngal mRNA determined by TaqMan^®^ analysis as described in Materials and Methods. The internal standard used was 18 s ribosomal RNA. (**C**–**E**) Representative Western blot (**E**) and quantification of all Western blot results from kidney lysates employing antibodies for KIM-1 (**C**) and NGAL (**D**). Data are shown as box plots with a line at the median and whiskers from minimum to maximum value; *n* = 5–9; n.d. = no protein band detected; n.s. = not significant; * *p* ≤ 0.05; ** *p* ≤ 0.01 as indicated.

Kidney damage was further quantified by looking at the expression of kidney injury markers (Figure 3A–E). Kidney injury molecule-1 (KIM-1) and neutrophil gelatinase-associated lipocalin (NGAL) protein expression was analyzed by Western blot, and the corresponding mRNA levels were analyzed via RT-PCR (TaqMan^®^). Expression of KIM-1 was clearly, but not in all cases, significantly lower in knockout mice on mRNA and protein levels compared to wild-type mice (Figure 3A,C). NGAL expression levels of knockout mice were also significantly lower after 7 days on mRNA levels and after 14 days on protein levels compared to wild-type mice (Figure 3B,D). No expression of KIM-1 and NGAL were detected in healthy mice, while in all diseased mice, a strong upregulation was observed (Figure 3C,D).

### 2.4. Inflammatory Cytokine Expression Was Reduced in S1P_5_ Knockout Mice after Disease Induction

**Figure 4:**
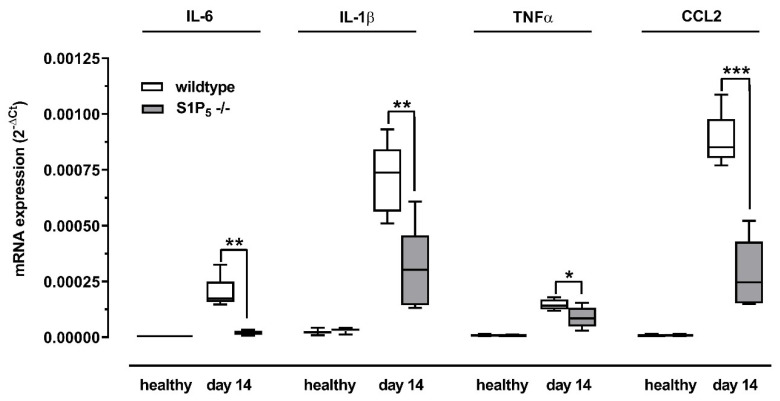
Gene expression of proinflammatory cytokines. Expression levels of genes were compared between wild-type and S1P_5_ knockout mice (S1P_5_^-/-^) without and with an adenine-rich diet for 14 days. Expression levels of Il-6, Il-1β, Tnfα, and Ccl2 mRNA were determined by TaqMan^®^ analysis as described in Material and Methods. The internal standard used was 18 s ribosomal RNA. Data are shown as box plots with a line at the median and whiskers from minimum to maximum value; *n* = 5–9; * *p* ≤ 0.05; ** *p* ≤ 0.01; *** *p* ≤ 0.001 as indicated.

Inflammatory cytokine expression in whole kidney homogenates was analyzed using RT-PCR. Ccl2, Il-6, Tnfα, and Il-1β mRNA expression were induced by an adenine-rich diet in all mice. However, expression was significantly lower in S1P_5_^-/-^ mice compared to wild-type mice for all mentioned cytokines after 7 days except for IL-1β (data not shown) and 14 days (Figure 4).

### 2.5. Similar Numbers of Renal Mononuclear Phagocytes in Kidney Cortex of Wild-Type and Knockout Mice

**Figure 5:**
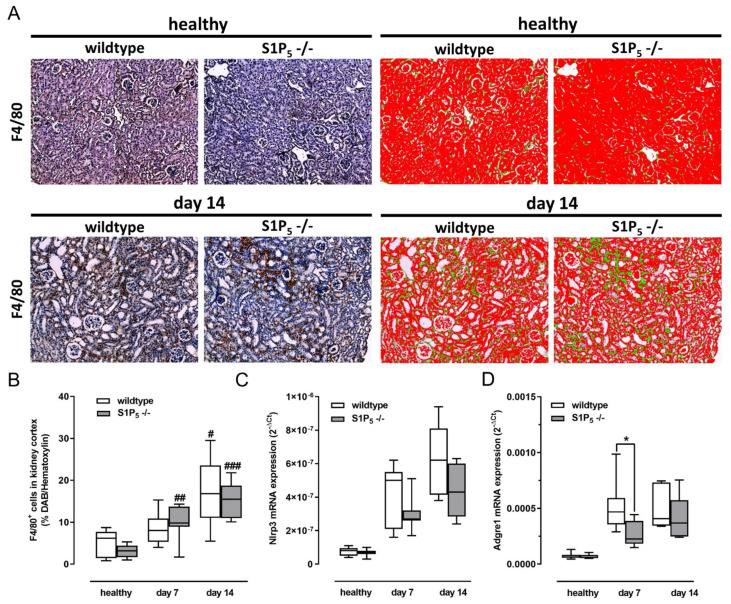
Presence of renal mononuclear phagocytes and expression of inflammasome component in kidney cortex. Expression of markers of mononuclear phagocytes were compared between wild-type (C57BL/6J) and S1P_5_ knockout mice (S1P_5_^-/-^) without (healthy) and with adenine-rich diet for 7 and 14 days. (**A**) Paraffin-embedded kidney cortex sections were subjected to immunohistochemical staining for F4/80 expression with DAB (brown) and counterstained with Mayer’s hematoxylin solution (blue). Algorithm-based detection of the staining was used to visualize and quantify the area covered by diaminobenzidine (green) and hematoxylin (red). (**B**) Median DAB area/hematoxylin area ratio was determined for up to 30 single pictures per tissue section, and the medians of all sections per group are shown here. (**C**,**D**) Expression levels of Nlrp3 and Adgre1 (also known as Emr1) mRNA were determined by TaqMan^®^ analysis as described in Materials and Methods. The internal standard used was 18 s ribosomal RNA. Data are shown as box plots with a line at the median and whiskers from minimum to maximum value; *n* = 5–9; * *p* ≤ 0.05 as indicated; # *p* ≤ 0.05; ## *p* ≤ 0.01; ### *p* ≤ 0.001 compared to the respective healthy controls.

Moreover, cells in the whole cortex region of each kidney were stained for F4/80 expression as a marker for mononuclear phagocytes, and the diaminobenzidine (DAB)-stained area was measured with the help of the tissue imaging programs Vectra^®^, Phenochart^®^ and inForm^®^ (PerkinElmer) (Figure 5A). The DAB-stained area, representing F4/80^+^ cells, was measured and visualized by a green color for each picture of kidney section. A Red color represents the area covered by hematoxylin stain and thereby the area covered by all cells, excluding tissue-free spots in the picture, to allow calculation of the ratio of F4/80^+^ cells to all cells in the kidney section. The presence of F4/80^+^ cells was increased in diseased kidneys but was not different between wild-type and S1P_5_^-/-^ mice (Figure 5B). In line with this finding, RT-PCR measurements of the gene that codes for F4/80, the adhesion G protein-coupled receptor E1 (Adgre1, also known as Emr1), and inflammasome component nucleotide-binding oligomerization domain, leucine-rich repeat, and pyrin domain containing 3 gene (Nlrp3), which codes for a protein part of the inflammasome that is activated by ingesting mononuclear phagocytes, were also similar in expression between both genotypes (Figure 5B,C). On the other hand, inflammatory cytokine release and NLRP3 inflammasome activation can also occur in renal tissue cells, and S1P_5_ activation in these cells could influence the inflammatory response [26,27]. 

However, mRNA levels of Adgre1 (gene of F4/80) were 6-fold higher in samples from wild-type mice after 7 and 14 days of an adenine-rich diet compared to healthy controls. At 7 days of adenine treatment, the mRNA levels were significantly higher in wild-type mice compared to knockout mice, and no significant differences were determined after 14 days. Gene expression of Adgre1 was 4-fold higher at day 7 and 6-fold higher at day 14 in samples from S1P_5_^-/-^ mice compared to the healthy controls. 

Since it has already been published that S1P_5_ is involved in the trafficking of NK cells [19], we also checked the mRNA expression of natural cytotoxicity-triggering receptor 1C (Ncr1) and killer cell lectin-like receptor subfamily B (Klrb1c), but we only found low levels and no significant differences between the two genotypes (Figure S1).

### 2.6. Fibrosis Was Attenuated in S1P_5_ Knockout Mice after Disease Induction

**Figure 6:**
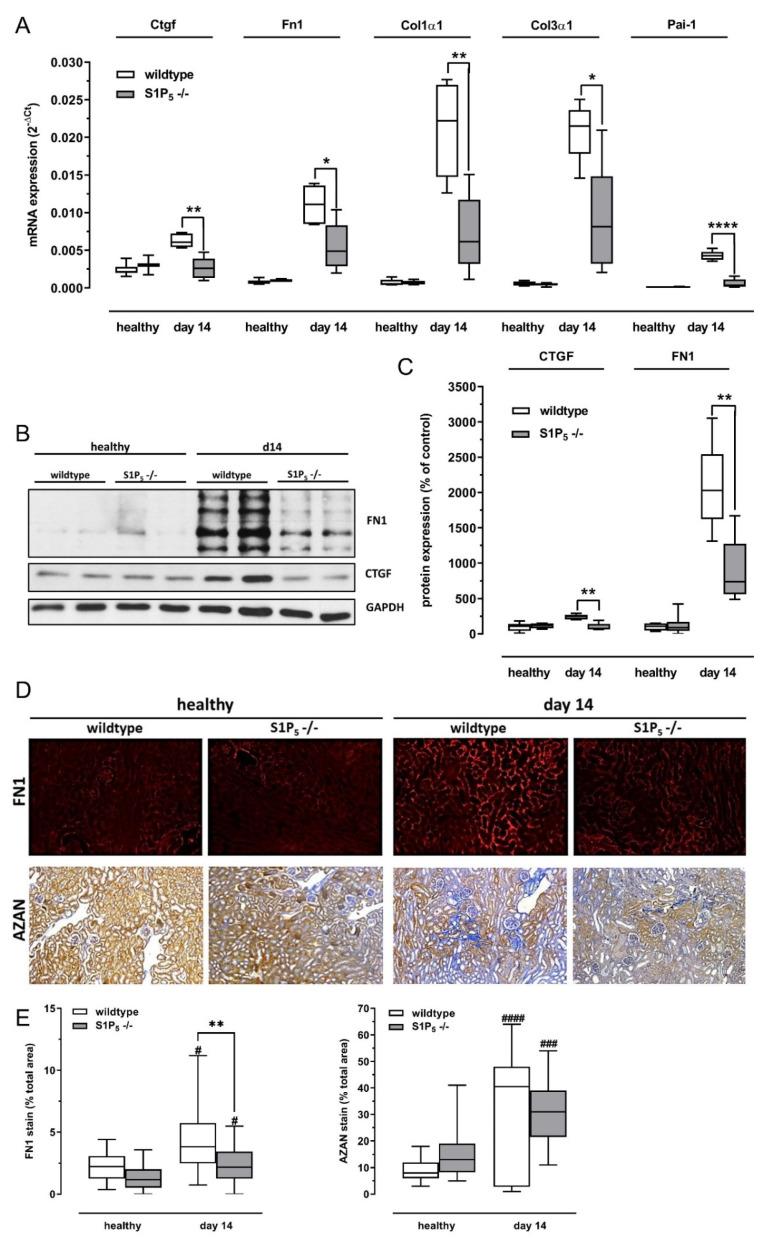
Gene expression and staining of fibrotic markers. (**A**) Expression levels of CTGF, Fn1, Pai-1, and Acta-2/αSMA mRNA were determined by TaqMan^®^ analysis as described in Materials and Methods. The levels were compared between wild-type (C57BL/6J) and S1P_5_ knockout mice (S1P_5_^-/-^) without (healthy) and with an adenine-rich diet for 14 days (d14). The internal standard used was 18 s ribosomal RNA. (**B**,**C**) Representative Western blot (**B**) and quantification results from all Western blots (**C**) of fibronectin 1 (Fn1) and connective tissue growth factor (CTGF) from whole kidney homogenates of wild-type (C57BL/6J) and S1P_5_ knockout mice (S1P_5_^-/-^) without (healthy) and with an adenine-rich diet for 14 days (d14). (**D**) Paraffin-embedded kidney cortex sections were subjected to immunohistochemical staining for fibronectin (FN1) and staining for cartilage and collagen fibers by AZAN dyes. Fibronectin staining is visible as a bright red color, and cartilage and collagen fibers are deep blue. (**E**) Semi-quantitative analysis of AZAN and FN1 staining (**D**), shown as % of total area (**D**). Wild-type (C57BL/6J) and S1P_5_ knockout mice (S1P_5_^-/-^) without (healthy) and with an adenine-rich diet for 14 days (d14) were compared. Data are shown as box plots with a line at the median and whiskers from minimum to maximum value; *n* = 5–9; * *p* ≤ 0.05; ** *p* ≤ 0.01; **** *p* ≤ 0.0001 as indicated. # *p* ≤ 0.05, ### *p* ≤ 0.001, #### *p* ≤ 0.0001 as compared to the respective healthy controls.

The expression of several markers of fibrosis was induced by the adenine-rich diet. In line with the results on inflammation and tissue damage, fibrosis was attenuated in S1P_5_^-/-^ mice compared to wild-type mice. RT-PCR analysis shows significantly lower expression levels of connective tissue growth factor (CTGF), fibronectin 1 (Fn1), plasminogen activator inhibitor-1 (Pai-1, official name SERPINE1), and alpha smooth muscle actin (Acta-2/αSMA) after 14 days of an adenine-rich diet (Figure 6A). CTGF and fibronectin expression were also investigated on a protein level by Western blot, and densitometric quantification revealed a significantly lower expression of these markers of fibrosis compared to wild-type mice after 14 days of an adenine-rich diet (Figure 6B,C). Furthermore, immunohistochemical staining was performed to assess fibrosis in wild-type and knockout mice after adenine treatment. Knockout mice showed less fibrosis compared to wild-type mice as seen by Fibronectin 1 and connective tissue expression in kidney sections after staining with corresponding antibodies, AZAN, and Sirius Red (Figure 6D). The semi-quantitative analysis of Fibronectin 1 staining indicates a significantly lower level of fibrosis in knockout mice compared to wild-type mice after 14 days (Figure 6E). We observe the same tendency in AZAN staining, but the difference between knockout and wild-type mice after 14 days was not significant in the semi-quantitative analysis (Figure 6E). 

## 3. Discussion

Here, we employed a mouse model of tubulointerstitial fibrosis to elucidate the role of S1P_5_ in kidney diseases. We found that plasma creatinine levels rose in wild-type mice after 1 or 2 weeks of an adenine-rich diet, while levels remained significantly lower in knockout mice. Serum or plasma creatinine is a broadly employed marker for the loss of renal function [28]. In addition, analysis of renal cortex samples subjected to PAS staining revealed that there was less tissue damage in S1P_5_^-/-^ mice compared to wild-type mice. Furthermore, we looked for the expression of KIM-1 and NGAL, which are considered to be early markers for acute kidney injury since they can be found in urine before an increase in serum creatinine occurs and are not expressed in healthy kidneys [29,30,31,32,33]. It is assumed that KIM-1 is expressed in response to the loss of cell–cell adhesion and prior to cell death [34]. NGAL is expressed in response to kidney injury in order to prevent apoptosis of renal tubular cells [35,36,37]. KIM-1 and NGAL were detected in kidney samples of all diseased mice, and the median levels of KIM-1 and NGAL were lower in knockout mice compared to wild-type mice. Taken together, S1P_5_^-/-^ mice are clearly less prone to sterile, inflammation-induced kidney damage.

Since the adenine-rich diet leads to the deposition of crystals within the kidney tissue, we analyzed the severity of inflammation in the kidneys. The expression of proinflammatory cytokines in kidney homogenates of diseased S1P_5_^-/-^ mice was significantly lower compared to wild-type mice. Since these cytokines can be released by infiltrating or proliferating immune cells, we stained for renal mononuclear phagocytes, which comprises macrophages and dendritic cells [38]. It was shown that renal mononuclear phagocytes are present in large numbers in inflamed kidneys and play an important role in disease progression [39,40,41,42]. Moreover, S1P_5_ knockout animals lack peripheral Ly6C^−^ monocytes, which could be precursors for tissue-resident macrophage populations [18,43]. The inflammatory Ly6C^+^ monocytes, in contrast, do not express S1P_5_ [18]. In the tissue of diseased wild-type and knockout mice, we found a high count of F4/80^+^ cells, a major marker of renal mononuclear phagocytes [38,44]. F4/80^+^ expression by antibody staining was significantly higher after 14 days of adenine treatment in S1P_5_^-/-^ and wild-type mice. In line with this, the mRNA level of Adgre1 (gene of F4/80) was 6-fold higher after 14 days of adenine-rich diet in samples from wild-type mice as well as from knockout mice without a difference between the genotypes. 

In contrast, mRNA expression of proinflammatory cytokines, such as interleukin-6 (IL-6) and tumor necrosis factor alpha (TNFα) were significantly lower in tissue of S1P_5_^-/-^, and these cytokines can be released by macrophages in response to inflammatory stimuli [45]. In addition, the mRNA level of monocyte chemoattractant protein-1 (MCP-1/CCL2) was also significantly lower in kidney homogenates of knockout mice although the number of F4/80^+^ cells was not lower in S1P_5_^-/-^ mice. The difference in cytokine release could be due to a different activation status of macrophages or other immune cells. However, cytokine release could also be different from renal mononuclear phagocyte counts because cells of the tubular system are also able to release these cytokines [26]. 

There is not much known yet about the role of sphingosine 1-phosphate receptor 5 (S1P_5_) in (differentiated) monocytes, and it seems that this receptor is only expressed on specific subtypes [18,46,47]. It was reported that antagonizing S1P_5_ with suramin improved phagocytosis in alveolar macrophages [47]. Inflammasome activation via phagocytosis of crystals in kidney tissue boosts inflammation and leads to the secretion of IL-1β [48,49]. However, mRNA expression of the nucleotide-binding oligomerization domain, leucine-rich repeat and pyrin domain containing 3 (Nlrp3), a component of inflammasome, was as strong in knockout as in wild-type samples. In contrast, IL-1β mRNA levels were not different between the two genotypes after 7 days but were more than doubled in wild-type samples after 14 days of adenine treatment. It was reported that sphingosine, but not S1P, was able to act as a danger-associated molecular pattern (DAMP) and activate NLRP3-inflammasome and the subsequent IL-1β release from macrophages [50]. Furthermore, it was shown in another study of adenine-induced renal fibrosis that treating mice with an inhibitor of NLRP3 activation, as well as employing NLRP3-deficient mice, reduced fibrosis [51]. Therefore, further studies need to be performed to investigate whether inflammasome activation due to phagocytosis is indeed impaired.

S1P was shown to be a chemoattractant for primary human monocytes and macrophages in vitro and that employing the S1P receptor functional antagonist fingolimod reduced not only lymphocyte but also macrophage infiltration in acute anti-thy1 mesangio-proliferative glomerulonephritis [52,53]. However, the immunosuppressive effect of the functional S1P receptor antagonists fingolimod and siponimod were attributed to the internalization and degradation of S1P_1_ [13,54,55,56,57]. These findings agree with our data showing no difference in immune cell infiltration caused by S1P_5_ deficiency although, potentially, subsets such as tissue-resident memory T cells were not specifically assessed [58]. Nevertheless, both of these drugs, which are approved for the treatment of multiple sclerosis, also have a high affinity for S1P_5_ [13,59,60].

S1P_5_ was reported to be critical for natural killer cell trafficking in vivo and migration in vitro but to have no effect on T cell migration [61,62]. Indeed, we also found no difference in T cell populations in the analyzed kidney homogenates. On the other hand, a difference in NK cell infiltration was not observed either. This might, however, be a result of lacking NK cell infiltration in general, since total numbers of NK cells were very low in all kidney samples and were not increased in diseased kidneys.

The mRNA and protein expression of the profibrotic cytokine CTGF was not induced in diseased knockout mice, while it was upregulated in diseased wild-type mice compared to healthy mice. Furthermore, the extracellular matrix protein fibronectin was significantly reduced at the mRNA and protein levels in diseased knockout mice compared to wild-type mice, and the same was seen for several other markers of fibrosis on the mRNA level. The difference could, however, be due to the milder tissue damage in knockout mice.

Among other proteins, S1P_5_ can couple to G_12/13_ and also activate c-Jun amino terminal kinase signaling pathway (JNK) [17,63]. It was shown that S1P-mediated activation of G_12/13_ and subsequent activation of RhoA and Rho kinases induces proinflammatory factor expression by macrophages [64]. In addition, there are several publications on proinflammatory and profibrotic factor expression mediated by G_12/13_ in renal cells and kidney disease [65,66,67]. Also, activation of the JNK pathway promotes renal fibrosis as reviewed by Grynberg et al. [68] and JNK becomes activated in tubulointerstitial cells in diseased renal tissue [69]. Taken together, there are several indications for a proinflammatory and profibrotic effect of S1P_5_ in renal disease that support our findings of an improved outcome for S1P_5_^-/-^ mice in adenine-induced tubular injury.

Nevertheless, more experiments are needed to elucidate the mechanisms behind the anti-inflammatory effects of S1P_5_ deficiency. Here, we showed that S1P_5_ seems to be an interesting target for the treatment of renal fibrosis or even inflammation, especially since drugs for the pharmacological modulation of S1P_5_ are already commercially available. A-971432 is a specific agonist of S1P_5_ provided by AbbVie, and siponimod (BAF312) from Novartis targets S1P_1_ and S1P_5_ and was approved in the USA for the treatment of secondary progressive multiple sclerosis [60,70], while other agents with S1P_5_ engagement may also be relevant (i.e., ozanimod, etrasimod) [13]. These and other agents may provide near-term assessments of new therapeutic strategies to treat chronic kidney diseases. As with all drugs, potential side effects mediated via S1P_5_ blockade in non-target organs may occur and need to be carefully evaluated. However, in our study, we observed no difference in phenotype and behavior of healthy S1P_5_^-/-^ mice compared to healthy wild-type mice. 

Taken together, we can present here for the first time that S1P_5_ has a distinct impact on the pathological outcome in sterile, inflammation-induced kidney disease. In addition, our study provides the basis for future studies and gives an incentive to unravel the specific interactions of S1P_5_.

## 4. Materials and Methods

### 4.1. Animal Model and Sample Collection

The S1P_5_^-/-^ mouse strain was generated [19] and kindly provided by the Scripps Research Institute (La Jolla, CA, USA). C57BL/6J mice were used as the respective wild-type controls. Male S1P_5_^-/-^ and wild-type mice were fed ad libitum with water and an adenine-rich diet (0.25% adenine, Altromin, Lage, Germany) for either 7 or 14 days (*n* = 5–7). Untreated, age-matched, male S1P_5_^-/-^ and wild-type mice were used as healthy controls (*n* = 9). All animals were housed under pathogen-free conditions in secluded ScanTainers in the animal housing facility of the Goethe University Hospital. For sampling, mice were anesthetized and sacrificed, and kidneys were collected and prepared for further analyses as described. No mice died until they were sacrificed at the end of the experiment. Kidneys were snap-frozen in liquid nitrogen immediately after sampling and stored at −80 °C until further analyses or bisected and stored in 10% buffered formaldehyde solution at 4 °C before paraffin-embedding. Blood was collected in EDTA tubes and centrifuged two times at 750× *g* (4 °C) for 5 min each to separate plasma. Snap-frozen organ samples and plasma samples were stored at −80 °C until further analyses. 

### 4.2. Enzyme-Linked Immunosorbent Assay (ELISA)

Creatinine levels in plasma were measured with a StressXpress^®^ Creatinine Serum Detection Kit following the instructions in the manual (StressMarq Biosciences Inc., Victoria, BC, Canada).

### 4.3. RNA Extraction and Real-Time PCR Analysis

For RNA extraction, deep-frozen kidney samples were homogenized in a Micro-Dismembrator S (Sartorius Stedim Biotech GmbH, Göttingen, Germany) at 3000 rpm for 30 s and resuspended in 1 mL of TRIZOL™ reagent (Sigma-Aldrich, Steinheim, Germany). RNA was extracted according to the manufacturer’s protocol and used for reverse transcriptase-polymerase chain reaction (RT-PCR) (RevertAid™ first-strand cDNA synthesis kit, Thermo Fisher Scientific, Darmstadt, Germany) using a random hexamer primer for amplification. Real-time PCR (TaqMan^®^) was carried out with the Applied Biosystems 7500 Fast Real-Time PCR System. TaqMan^®^ gene expression assays and PCR Low Rox Mix were obtained from Thermo Fisher Scientific (Darmstadt, Germany). The TaqMan^®^ gene expression assays are listed in the Appendix A, Table A1 (Thermo Fisher Scientific, Darmstadt, Germany). The threshold cycle (Ct) was calculated by the instrument’s software (7500 Fast System SDS Software version 1.4). Analysis of the relative mRNA expression was performed using the ΔΔCt method. Eukaryotic 18S ribosomal RNA (Thermo Fisher Scientific, Darmstadt, Germany) served as a reference for normalization.

### 4.4. PCR and Agarose-Gel-Electrophoresis

The PCR reaction was performed with an annealing temperature set to 60 °C, using custom-made S1P_5_ forward (5’GTGACTTGTGATGTGAGCTG3’) and reverse (5’CTTGTCTCCTCCCATTCTCC3’) primer obtained from Eurofins Scientific (Luxemburg). PCR products were examined by electrophoresis using a 3% agarose gel.

### 4.5. Western Blot Analysis

For Western blot analyses, deep-frozen kidney samples were homogenized in a Micro-Dismembrator S (Sartorius Stedim Biotech GmbH, Göttingen, Germany) at 3000 rpm for 30 s and resuspended in lysis buffer (50 mM Tris-HCl, pH 7.4, 150 mM NaCl, 10% glycerol, 1% Triton X100, 2 mM EDTA, 2 mM EGTA, 40 mM β-glycerophosphate, 50 mM sodium fluoride). Equal amounts of protein were separated by SDS-PAGE, transferred to nitrocellulose membrane, and utilized in Western blot analysis using antibodies as indicated. Antibody against FN1 (ab2413) was obtained from Abcam (Cambridge, UK). The GAPDH (sc-20357) and CTGF (sc-14939) antibodies came from Santa Cruz Biotechnology (Heidelberg, Germany). HAVCR/KIM-1 (#AF1817) and Lipocalin-2 (#AF1857) antibodies were from R&D Systems (Minneapolis, MN, USA). Secondary antibody donkey anti-goat IgG, HRP conjugate (#AP180P) was obtained from EMD Millipore (California, CA, USA) and secondary antibody donkey anti-rabbit IgG, HRP conjugate (#NA934) was obtained from Sigma-Aldrich (Munich, Germany).

### 4.6. Histological Staining and Analysis

Paraffin-embedded renal sections (3 μm) were deparaffinized and stained with the periodic acid-Schiff reaction (PAS). Counterstaining was performed with Mayer’s hematoxylin (Sigma-Aldrich, Germany). The tissue was analyzed with the help of light microscopy by three blinded observers to assess tubular damage. The following tubular injury score, comprising 5 categories, was deployed: 0: 0%; 1: ≤25%; 2: >25% to 50%; 3: ≥50% to <75%; 4: ≥75% of the area displaying tubular damage. Median score of all renal sections from an individual kidney was calculated and presented in a pie chart.

Immunohistochemistry for the detection of F4/80 and fibronectin was performed on 3–4 µm thick paraformaldehyde-fixed, paraffin-embedded kidney sections. Sections were deparaffinized, washed, microwave-treated in citrate buffer (0.01 M/0.05% Tween-20, pH 6) and blocked for 30 min with Protein Block Serum-Free solution (Agilent, California, USA). Incubation with the primary antibody rat anti-mouse F4/80 (Serotec) was performed overnight at 4 °C. After blocking endogenous peroxidase and incubation with respective antibodies, sections were developed with the Diaminobenzidine Substrate Kit (Vector Laboratories, Burlingame, CA, USA). Counterstaining was performed with Mayer’s hematoxylin (Sigma-Aldrich, Munich, Germany). F4/80 staining in the cortex region was visualized and quantified automatically based on an algorithm with the help of the Vectra^®^ Polaris™ Automated Quantitative Pathology Imaging System in addition to tissue-imaging programs Vectra^®^, Phenochart^®^, and inForm^®^ (PerkinElmer Inc., Waltham, Massachusetts, USA).

To assess extracellular matrix deposition, slides were incubated with an anti-human fibronectin antibody (raised in rabbit, also recognizing mouse and rat FN, 1:2000 in Dako Antibody Diluent S3022, Gibco, A101, no longer available) for 1 hour at room temperature and overnight at 4 °C. After washing with TBS/0.05% Tween-20, signal detection was carried out using a Cy3-labled secondary antibody (goat anti-rabbit, Dako Antibody Diluent, Jackson Immuno-Research 1:500). Corresponding amounts of normal rabbit serum were used as negative controls. Staining results were analyzed and documented using a Leica DFC-480 camera connected to a Leica DM–RB microscope. AZAN staining was performed according to Geidies to investigate fibrosis in mouse renal tissue [71]. Slides were incubated for 30 min with phosphotungstic acid, stained for 15 min with aniline blue and orange G solution, and dehydrated with isopropyl alcohol and xylol before mounting.

### 4.7. Statistical Analysis

Statistical analysis was performed in GraphPad Prism (version 9.3.0, GraphPad Software Inc., San Diego, CA, USA). Differences between nonfibrotic and fibrotic samples were analyzed by paired t-test if the values of all analyzed groups passed a Kolmogorov–Smirnov normality test. Otherwise, values were analyzed by Mann–Whitney U test. Differences of *p* ≤ 0.05 were considered to be statistically significant. 

## Supplementary Materials

The following supporting information can be downloaded at: https://www.mdpi.com/article/10.3390/ijms23073952/s1.

## Appendix A

**Table A1:** Genes and Assay IDs used for TaqMan^®^ analyses.

Gene	Abbreviation	Assay ID
adhesion G protein-coupled receptor E1	Adgre1/Emr1	Mm00802529_m1
Connective tissue growth factor	Ctgf	Mm01192933_g1
Fibronectin 1	Fn1	Mm01256744_m1
Kidney injury molecule-1	Kim-1	Mm00506686_m1
Neutrophil gelatinase-associated lipocalin	Ngal/lcn2	Mm01324470_m1
Nucleotide-binding oligomerization domain, leucine-rich		
Repeat and pyrin domain-containing 3 gene	Nlrp3	Mm00840904_m1
Natural cytotoxicity triggering receptor 1C	Ncr1	Mm01337324_g1
Killer cell lectin-like receptor subfamily B	Klrb1c	Mm00824341_m1
Serpine1/plasminogen activator inhibitor-1	Pai-1	Mm00435858_m1
Interleukin-6	Il-6	Mm00446190_m1
C-C motif chemokine ligand 2	Ccl2	Mm00441242_m1
Interleukin-1 beta	Il-1β	Mm00434228_m1
Sphingosine 1-phosphate receptor 5 Tumor necrose factor alpha	S1P_5_ Tnfα	Mm02620565_s1 Mm00443258_m1
