# Supplementary material for: Sphingosine 1-Phosphate Receptor 5 (S1P5) Knockout Ameliorates Adenine-Induced Nephropathy
Source: Int J Mol Sci. 2022 Apr 2;23(7):3952. doi: 10.3390/ijms23073952 (PMC8999641; doi:10.3390/ijms23073952)
Supplement: Supplementary file 1 [file ijms-23-03952-s001.zip › ijms-1596240-supplementary.pdf]

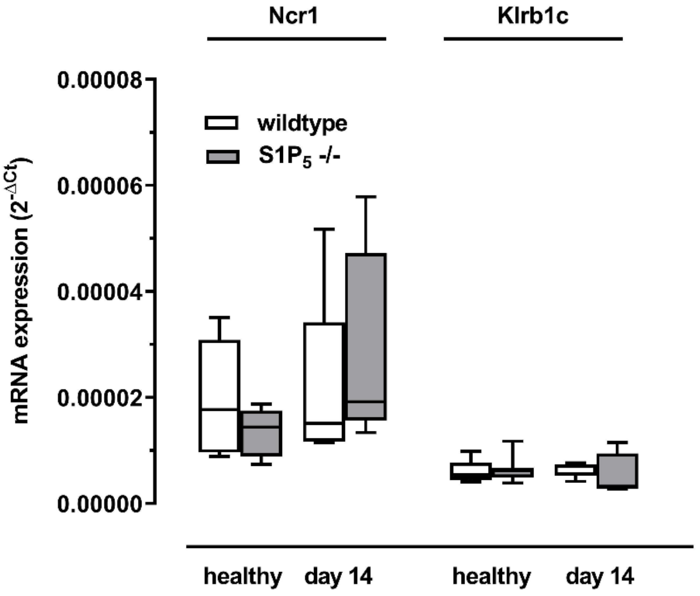

Figure S1. Expression levels of Ncr1 and Klrb1c were determined by TaqMan® analysis as described in Materials and Methods. The levels were compared between wild-type (C57BL/6J) and S1P5 knockout mice (S1P5-/-) without (healthy) and with an adenine-rich diet for 14 days (d14). The internal standard used was 18 s ribosomal RNA.
